# Veterinary Jurisprudence1Read before the twenty-seventh annual meeting of the United States Veterinary Medical Association, at Chicago, September 17th, 1890.

**Published:** 1890-10

**Authors:** A. Liautard


### VETERINARY JURISPRUDENCE.[1]

By Prof. A. Liautard.

There are two things connected with the circumstances of the present occasion which I cannot fully account for.

The first is that I should be selected by your worthy secretary, Dr. Hoskins, to take so prominent a part in your proceedings, and the second is that I should presume so far as to consent to

[1] Read before the twenty-seventh annual meeting of the United States Veterinary Medical Association, at Chicago, September 17th, 1890.

appear before this grand annual reunion of members of our noble profession—this great gathering of veterinarians from nearly every portion of the land—in the character of a conveyor of instruction or the originator of measures or policies of action. To meet with you, gentlemen of the West—you to whom I was wholly a stranger when with others I came to this great and wonderful city to unite our counsels for perfecting the establishment upon permanent foundations of the United States Veterinary Medical Association—was, I am afraid, a great presumption on my part. But the good of the profession was held prominently in view; the welfare of our Association was at stake, and the prospect of solidifying a national feeling of brotherhood and a friendly professional union were all to be considered, and I felt that when thus requested to aid in your discussion my silence could hardly be justified.

And do not these objects furnish a sufficient apology for my confidence in coming before you, and will they not also constitute a sufficient plea for the indulgence with which, I doubt not, you will accept my remarks, even if they should, haply, miss your approbation and fall short of your expectations? Upon this assumption I proceed.

Gentlemen, when we quietly reflect upon the duties and obligations of the veterinary surgeon, we can hardly avoid being impressed with the fact that ours is not simply a single calling, but is largely multiform in its character, and possesses some very peculiar features; and that these are in some instances of such a nature that it is a wonder that the value of the veterinarian and of the services which he renders to the public should not have been at best sufficiently appreciated to secure for him the social, official and legal standing which he may justly claim for himself.

Many duties are imposed upon the veterinarian which are avoided or escaped by his brethren in human medicine, who has it in his power, in many cases, to assume the title of specialist and to enter upon the peculiar studies pertaining to the rôle he has chosen, while, on the other hand, the veterinarian, generally speaking, is not only expected to be a competent general practitioner, and even a surgeon, but has often to add to these the functions of an obstetrician, and to that again even the duties of the general sanitarian. Thus, he must be now an equine pathologist; then an expert in cattle diseases; next, even a connoisseur in diseases of the lower animals, including dogs and birds ; all this, if not more, but he must, in order to supplement these various functions and capacities, and others which might undoubtedly be specified, stand ready to encounter the risk of burning his fingers and jeopardizing his reputation by handling points in law and becoming entangled in matters of jurisprudence and questions of traffic. The beginning of his trouble here is the moment when he is called upon to give his professional opinion in connection with the sale and purchase of domestic animals, principally in the sale of horses.

And this is the subject I have chosen as the topic of my remarks on the present occasion.

The decision of the question of the soundness of a horse, or, in other words, his value when offered for sale or sought for purchase, is one of the most important of the duties devolving on the veterinarian. As affecting the seller or the purchaser of the animal, it is of course the first in importance, and it is one which is constantly coming before the veterinarian for the exercise of his skill and trial of his integrity, and I may add, his courage. Every day animals are brought to him for examination and judgment, and he is called upon for written certificates as to their condition and soundness, and it is usually his verdict which determines the question of sale or no sale. The responsibility which is thus laid upon his shoulders may be, and is in fact, sometimes enormous, and no man can count the damage he may incur from an unacceptable decision, *pro* or *con*, however honest, when the result of such an opinion may spoil a good trade for one man, or involve another in a bad bargain.

Gentlemen, in the performance of this part of our professional duty there is no possibility of escape or of chance for the concealment of an opinion ; it must be stated in black and white, and whatever your judgment may be, it is most likely, in a majority of cases, to be a condemnation of the animal.

I said "enormous" responsibility. Yes, not only in a professional sense, but a matter of social involvement. Standing between the seller and the buyer, our verdict must be candid and true. Upon no one else can reliance be placed for a correct, fair and honest judgment. No man must buy your opinion ; no partiality must influence your verdict ; you are nobody's friend, but a judge standing impartially between two parties, one with something to sell and one wanting to buy something; and you are to pronounce upon the quality of an article of merchandize, without considering who may be benefited or who may be injured by your opinion.

Yes, it is a trust of which we veterinarians ought to be proud one of which we must acquit ourselves with the most delicate fidelity, with the nicest integrity of purpose, with the most scrupulous carefulness; one, indeed, which I believe places us in the first rank amongst useful citizens, by reason of an intimate connection which is thus established between ourselves and the courts of justice. It is a trust, also, which is far from being always a pleasant one to exercise, for it may often bring us within the scope of the threatened vengeance of violent and unscrupulous individuals, as well as the temptations of pecuniary offers, and may all too frequently place us between friends and relatives, with, we know not, what hazard of planting estrangement and emnity; yet with but one line of conduct for us to follow, in answering the single question referred to us for solution, to wit: "*Is this horse sound or unsound.*"

The fact that this is in an important sense an English-born nation, with a congenital and ineradicable spirit of traffic in our constitution, or that at least most of our antecedents and customs are English, accounts for the fact that many of our laws are either of English origin or are conformed to English precedents or spring from English usages, and those which regulate the trade in horses or relate to questions of property in the animal, are far from being exceptions to this rule. It is for this reason that, following English traditions, we are guided to a great extent in our examination and the conclusion we arrive at in horse trades by what we find laid down in British works on this subject, and principally in those of Oliphant.

What is soundness? is indeed, not a question which can always be answered correctly off-hand. If it must mean a *perfect* horse—perfect in form and wholly free from blemish or disease, it will undoubtedly be as difficult, perhaps, as impossible a task to find such an animal as it would be to find a literally perfect human being. But this cannot be the true meaning of the definition of soundness. The term certainly cannot mean literal perfection; but where then, it may be asked, can it be said that perfection ends and imperfection begins ; at what point or line will soundness become merged ?

The definition of J. Stewart, who says: "that a horse is sound when he has no disease about him, nor any effect of disease that renders, or is likely at any future period to render him less useful than he would be without it," can scarcely be admitted, because it approaches too nearly the idea of literal perfection, especially when the author goes on and states further that a "horse may have disease about him and be sound ; he may at least have the effects of disease, have splints, bony or callous tumors, warts, specks on the eye, be blemished all over and still be a sound horse." I might ask what we would think here of a veterinarian certifying as sound an animal whose metacarpal regions were covered with splints, whose coronets were enlarged with large ringbones, whose eyes contained speck cicatrices of keratoma, and whose coarse hocks were scarred with the cicatrices of actual line and point cauterization ?

Oliphant tells us that "a horse free from hereditary disease, being in the possession of his natural and constitutional health, and having as much bodily perfection as is consistent with his natural formation, can be certified of being sound.

No doubt this is a much better definition that that of Percivall, who says that "any horse that is lame or has that about him which is likely, on work, to render him lame, is unsound," a definition which largely covers the subject of soundness so far as the function of locomotion is concerned.

But aside from all this, with us, the question must necessarily remain the same. Courts of law will differ in their opinions, and judges will express the varying ideas of various nations on the subject, and the announced results of experienced veterinarians will be too often treated with contempt, or ridiculed on account of difference of opinion, and their various interpretations of lesions and symptoms ; and to use the word of a learned judge in a case recently decided : "It is more and more wondrful to know how anyone could pretend to know what was soundness and what was not soundess."[1]

In the presence of such facts, gentlemen, facts with which I think you are all familiar, and in the light of such an experience as is common with us all, and of occurrences which I have wit-

[1] *Veterinary Record*, June 12th, 1890.

nessed too often in a long and extensive practice of this specialty, a doubt has often arisen in my mind whether we are doing right in conducting our examination as we do, and whether in granting our certificates as most of us do, we are dealing justly with all parties concerned, with our employer, with the dealer, and with the horse that has been brought before us. Are we dealing in a strictly legal sense with the inquiries made of us, and justifying fully the confidence lodged in our integrity and intelligence?

What, indeed, is asked of us by the gentleman who wishes to become the purchaser of an animal which, after satisfactory trial, he has selected as one fit to do his work and minister to his pleasure? The question is a simple one: "Is he sound?" and the answer ought to be equally simple. But how many horses can fulfil the conditions of an unqualified answer, even if literal perfection is not demanded? We may pass a horse because nothing but a small splint is visible, and yet in a few days he may become lame with it.

We may reject the next case because of a small exostosis on one hock, or a small spavin, and yet he may be purchased and may turn out a good and serviceable animal. Can we always distinguish, or are we to reject every animal which exhibits one or any of the ailments that we find in the true sense of the word to constitute unsoundness. To use the words of Dr. William Hunting, the editor of the *Veterinary Record*, "the want of a good definition renders the seller of a horse who gives a warranty, or *the veterinarian* who gives a certificate, liable to actions at law by the buyer whenever his new purchase goes wrong." And if this should be a correct view, it is easy to see in what a peculiar position we may find ourselves in some easily supposable cases. Let us suppose some condition in which an animal may be affected with some form of acute ailment, which has assumed a serious character, and may become incurable, or possibly prove fatal. Here is a patient, which at the time of examination, had but a slight discharge from one nostril, not very abundant; muco-purulent; inoffensive. We consider it a simple case of catarrh, and before many days have passed it proves to be merely the first indication of a diseased condition of a tooth, which may prove troublesome and refractory to treatment, and, perhaps, results finally in death. Again, assume an animal free from any blemish or unsound indication other than a slight increase of temperature, and, perhaps, a slight cough. He is bought, though sickly, and only a few days later dies from an attack of lung disease of an acute character, perhaps grafted on an organ already affected with chronic disease. Next in the category may come an animal in apparently perfect health, but which dies a few days after with colicky pains, due to calcareous formations in the intestines or a vesical calculus.

Similar cases almost without number might, if necessary, be cited in illustration of the point we are discussing. Are we to reject all these because, as vaguely and indefinitely expressed, they are "sick?" Yes; but what, then, of those with (perhaps latent) diseases which we have not been able to detect, or whose termination we have failed to foresee? If such as these are to be rejected for mere negative reasons and on hypothetical grounds, are we, again, doing justice to our calling, to our obligations and to all the three parties concerned? If not, I fear that the general investigations which constitute our so-called "examinations" are mere farcical and barren pretexts, and our certificates are worth less than the paper on which they are written, and can furnish no protection to the buyer, but, indeed, are rather detrimental to the honest dealer and unjust toward the good qualities of an animal which, though unsound, we know in many instances might prove to be a very useful servant, and fully compensate his purchaser, and perhaps might be turned against us in a court of justice.

If I am right, gentlemen, in suggesting such an interpretation of our position, and such a view of the performance of an examiner's duty, according to the English law of warranty, as well as the, at least possible, ill-effects of its application, it seems to me that the moment may not have been badly selected to consult with this assembly of veterinarians on the subject, and to ask for their opinion and their suggestions in respect to a remedy for the evils to which I have referred.

This is no new subject with me. Years have passed since its first presentation by myself in Boston, before this same Association. But we were young then—our membership was small—and to-day, when we have strengthened our standing by the accession of so many worthy and accomplished associates, I hope the matter will receive at your hands the attention and consideration to which it is so well entitled. And now, admitting the inefficiency of the old law, with its errors, the problem presents itself in two forms:

First, what better methods can be devised? and, secondly, if proposed, can they be made practicable and available, and if so, by what means?

Let us consider the first of these points, and to answer this it will be necessary to call your attention to foreign legislation on the subject, and to refer you to the laws which exist in various European States. Let us, for instance, consider the French law which, with some modification, however, we find re-enforced in other nations, including Belgium, Switzerland, and, I believe, Italy, and this may be called the Continental law. In this law the condition of soundness or unsoundness is not judged by the rules which prevail with us, although the method leads to the same results. But the point principally considered, is the effect of the unsound condition when it exists, so far as it bears upon the usefulness or the value of the animal. For example, the question is not so much whether an animal has any of the exostoses of the locomotory apparatus, or whether, when present, they interfere as a matter of fact with his working power, as by causing lameness, for example. It is not so much whether he is affected with disease, either acute or chronic, as whether, in a case of fatal termination, it existed previous to the purchase. It is not soundness as we understand it, concerning which the veterinarian is called to decide, but whether the animal has a "vice redhibitoire," which, at the time, interferes with its present and possibly future usefulness or value. By the term "vice redhibitoire," is understood a vicious and deficient condition, such as those which by law render the contract void, if the animal was warranted free from them. By this definition, you observe, this warranty is almost synonymous to our English warranty of soundness, which means free from diseases that constitute an unsoundness, while the other means free from any "vice redhibitoire," which have been specially designated.

To illustrate the first: An exostosis will not be a *vice redhibitoire* as long as it does not give rise to the characteristic lameness of those affections, "intermittent in character," either when warm or when cool.

A disease which proves fatal soon after, or within a few days of the purchase, will not authorize the repudiation of a bargain, unless it is proved that it existed before purchase, or was grafted on a chronic lesion previous to it. And it is thus that we find intermittent lameness and chronic affections of the lungs prominent among the "vices redhibitoires" of continental laws of warranty.

Considering now both what you will permit me to denominate the English and the French laws together, we shall no doubt find much similarity in the two cases mentioned. The result may be about the same, but how much simpler and more satisfactory otherwise, professionally considered, the latter, in view of the performance of the duties and the execution of the trust placed in our hands. To comply with our English laws we must certify to the existence of a condition which can hardly be found, either in man or horse—*perfection* in the abstract and literal sense of the word—and expose ourselves to no end of perplexity and trouble in the pursuit, while for the fulfilment of the more rational and practical provisions of the Continental laws, we are only required to detect within a certain period of time the presence of a few designated diseases, or the manifestations of a few definite symptoms. The English law obliges us to detect one of all diseases, the French code asks us to discover at the time of purchase or previously, or within a given period from the delivery, any one of the following diseases and pathological conditions, viz. :

Speaking at present for the horse only—periodic ophthalmia, glanders, farcy, immobility, pulmonary emphysema, intermittent lameness of long standing, intermittent hernia and chronic diseases of the thoracic organs, roaring, and some peculiar forms of cribbing.

And there is another point which I am sure will, in your estimation, count in favor of the Continental mode of examination. I refer to the time at our disposal for the completion of our task. Indeed, what time is allowed to us for the formation of our judgment, and for the preparation and the delivery of our certificate? A very sufficient time, as we all know. It may vary from a few minutes to perhaps a few hours. Is that sufficient? How in such a length, or rather such a "shortness" of time, are we to detect some of the special forms of disease, peculiar intermittent lameness, roaring, perhaps intermittent hernia, epilepsy, etc. In the European law from nine to thirty days, according to the "vice redhibitoire" looked for are allotted to the buyer, who thus has ample time to satisfy himself of the quality of his bargain, and thus also the veterinarian has a reasonable opportunity to detect an unsound condition, which, according to our English mode, becomes impossible.

In fact, Mr. President and gentlemen, it seems to me that in the superiority which I have endeavored concisely to point out in the European legislation on this subject, we are inevitably brought to the following conclusion :

Under a reformed regime, when it is once established, we shall have no more fear of contradictory certificates—one of them asserting soundness to-day, to be followed by another, affirming unsoundness four or five days later, and *vice versa*—no more danger of opposing opinions amongst veterinarians employed as experts who discredit one another, and the general profession likewise, by contrary announcements of the presence or the absence of morbid conditions ; no more decisions of judges in the courts of law directly in opposition to those of practising veterinarians ; and, above all, no more doubts, or at least a reform of opinion in respect to the supposed and generally imputed dishonesty of every man in the community who chances to have a horse for sale.

In respect to the consideration of the means by which such a law, after being properly framed, could be made practicable, I see no reason why such a thing might not be effected in this country as readily as it is in the various European States, if we will but bring ourselves to profit by their experience and follow their example in framing our laws and putting them in execution, as they have done and are doing.

But I have already held your attention too long, and the subject is so exhausted that I am almost tempted to ask you, paradoxical as it may seem in me, not to discuss it. I yet consider the matter to be one of such importance to all parties interested, and see so much injustice to be reformed, so much opportunity to do good both to buyers and sellers, and so fair an opening for securing a benefit to ourselves in the elevation of our profession, in the performance of this special duty of the examination of animals on purchase, that I can scarcely bring myself to suspend the discussion. I trust to your good nature to forgive the detention to which I have subjected you, and to attribute it wholly to my interest—which I am sure you share with me—in the subject which I have endeavered to illustrate.

